# A Narrative Review on Internet of Things and Artificial Intelligence for Poultry Production

**DOI:** 10.3390/ani16091285

**Published:** 2026-04-22

**Authors:** Anjan Dhungana, Bidur Paneru, Samin Dahal, Lilong Chai

**Affiliations:** Department of Poultry Science, College of Agricultural & Environmental Sciences, University of Georgia, Athens, GA 30602, USA

**Keywords:** cyber-physical systems, deep learning, poultry production, precision farming

## Abstract

Recently, poultry production has increased worldwide to address the increasing demand of affordable animal-sourced protein. To meet this requirement, poultry production operations have become more concentrated, introducing management challenges related to disease control, productivity, and animal welfare. However, manual flock monitoring and management have become impractical in such cases, creating a need for automatic data-driven management approaches. In this context, the Internet of Things (IoT) has emerged as a potential technological solution for continuous flock monitoring, data sharing, and decision-making. Despite this, its adoption in poultry production is limited compared with its widespread use in crop production, transportation, and manufacturing industrial sectors. Furthermore, advanced analytical techniques such as artificial intelligence (AI), applied to data gathered by IoT-enabled devices, have shown promising results by generating actionable information. Existing literature suggests that the integration of IoT and AI can address the major challenges associated with modern large-scale poultry production systems. While most applications remain at the research scale, such technologies have the potential for improving flock monitoring, enhancing productivity, and ensuring proper animal welfare. This narrative review examines the current state of IoT and AI based technologies, together or in part identifies the limitations, research gaps, and opportunities for future development.

## Simple Summary

With larger population, global demand for animal-based protein has increased rapidly, leading to larger and more intensive poultry production systems. As the flock size grows, it has become more difficult to monitor bird health, and environmental and welfare parameters using traditional manual observation. Inefficient monitoring can lead to delayed disease detection, reduce productivity, and negatively affect animal welfare. Devices that collect information from the farm can continuously record data on a large scale, and artificial intelligence can act upon such acquisitions to yield results that can help farmers make better management decisions. Studies suggest that using such technologies in poultry production is beneficial, however, their use in poultry farming is still limited compared to other industries. This review summarizes the current research on such technologies, and their specific applications in poultry production scenario, while also reflecting upon the challenges for their widespread adoption and future directions.

## 1. Introduction

The poultry industry is one of the fastest-growing animal production sectors and contributes significantly to 34% of the total global meat supply [1]. This large share is due to the affordability of chicken, the health advantages of white meat, and its versatility in culinary uses [2]. In the United States, poultry farms are becoming larger and more concentrated, with fewer farms managing a greater proportion of total production [3]. In 2024, US poultry farming produced about 9.4 billion meat chickens, and most of them were produced by the top five largest producers [4]. While such consolidation improves efficiency, it also increases the risks associated with disease outbreaks and complicates flock management. To manage the increasingly large operations and to ensure expected production, the use of new technologies has become a norm in the current scenario.

Recent breakthroughs in technologies have transformed agricultural production systems through the adoption of unmanned aerial vehicles (UAVs), sensor networks, and affordable high-performance computing (HPC). With the advancements in technology, various sectors have widely adopted the Internet of Things (IoT) for animal and environmental monitoring. IoT is a framework that interconnects devices to enable real-time data exchange and automation. To date, this system has been widely applied in animal husbandry, including cattle monitoring [3,5], and pig breeding and behavior recognition [6,7]. In poultry production, IoT has been explored for flock health monitoring [8], management [9], and behavior monitoring [10].

Interconnected systems like IoT enhance productivity and animal welfare, but inherent technological complexities make data management and practical implementation challenging. Since not all data collected at the production level holds equal informative value, effective data management is required to prevent unnecessary data transmission across the network, thereby reducing network load, costs of data transmission, and ensuring overall efficiency. Integrating artificial intelligence (AI) with IoT enhances data management and expands the capabilities of IoT systems by enabling efficient data analysis, pattern recognition, and decision-making based on sensor outputs [11]. This allows the IoT systems to not only collect the data but also interpret it, offering valuable information for early disease detection, welfare assessment, and flock management in poultry production.

Despite growing interest in IoT, the poultry-specific literature in this area remains fragmented, often separately focused on aspects such as environmental monitoring, health tracking, or resource use efficiency. Research addressing integrated systems that combine localized sensor networks, edge computing, and AI analytics is still limited. This combination is particularly important in commercial production systems, where massive volumes of video, audio, and sensor data must be processed efficiently. Edge computing devices can provide real-time analysis, reduce communication costs, and ensure continuity in areas with unstable connectivity. However, application of such devices in poultry systems has been underexplored. At the same time, small-scale poultry farms face restrictions in IoT adoption because of high equipment costs and limited technical expertise. Solutions tailored for affordability and scalability are needed to extend the benefits of IoT and AI beyond large commercial operations. Moreover, with the expansion of connected devices, comes growing concern about data security and privacy, necessitating robust frameworks for safe deployment [12,13].

**Figure 1:**
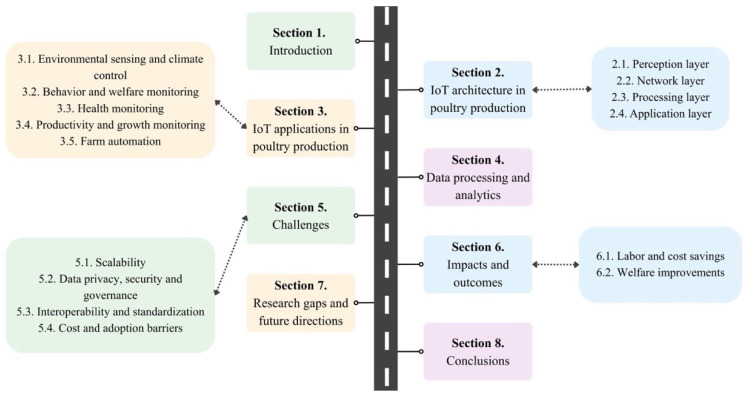
The structure of the review article.

This narrative review examines the current state of IoT and AI applications in poultry production, emphasizing their role together in improving productivity and welfare. It explores sensor technologies, data processing methods, and network architectures, highlighting opportunities for edge computing and localized systems. The paper also identifies challenges, including cost optimization, energy efficiency, and security, providing recommendations for future research. By situating poultry-specific systems within broader IoT frameworks, this review aims to guide the development of sustainable, scalable, and welfare-oriented technologies for the poultry industry. A detailed roadmap of this review is provided in Figure 1.

## 2. IoT Architecture in Poultry Production

**Figure 2:**
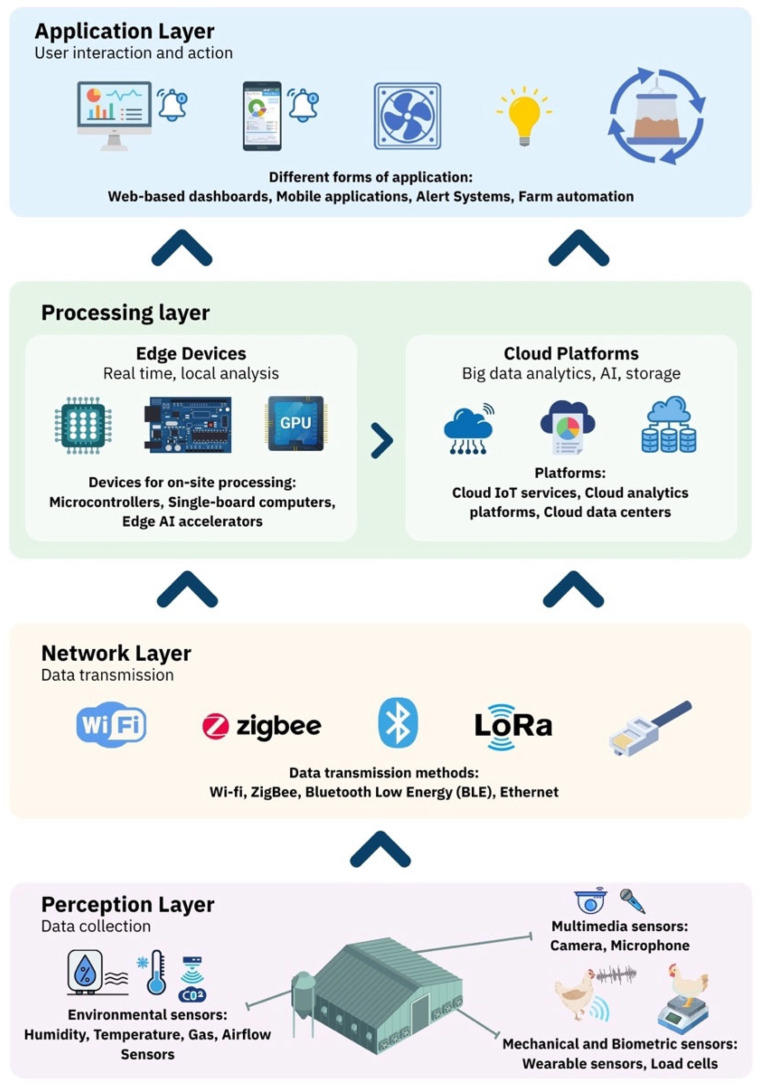
Four-layer Internet of Things (IoT) architecture for poultry farms.

In IoT systems, data flows through multiple layers starting from the sensors. In this review, we focus on a four-layer model consisting of the perception layer, network layer, processing layer, and application layer, as shown in Figure 2 to highlight the growing role of edge and cloud computing in poultry production.

### 2.1. Perception Layer

This layer encompasses all sensors and data acquisition devices, where environmental sensors (temperature, humidity, gas), and behavioral sensors (cameras, microphones, load cells, wearables) feed raw signals into the system [8,13]. These devices support continuous monitoring of microclimate and behaviors such as movement and feeding patterns and pecking or breathing. However, challenges like high cost, reliability under intensive production conditions, and scalability remain.

### 2.2. Network Layer

This layer transmits sensor outputs through wired or wireless systems. In poultry houses, both options are used depending on farm size and infrastructure. Wireless Sensor Networks (WSNs) such as Wi-Fi, ZigBee, and Bluetooth Low Energy (BLE) are common for short- to medium-range applications, while Ethernet supports more stable high-bandwidth connections. Each technology presents trade-offs: for example, Wi-Fi provides high data rates for video and audio but consumes more energy, while ZigBee and BLE are more energy-efficient but limited in range and throughput [11,14,15]. Hence, effective selection of technology in this layer is one of the most important aspects of IoT.

### 2.3. Processing Layer

Often referred to as the “edge layer,” this layer transforms raw sensor data into meaningful information. This layer performs data filtering, aggregation, temporary storage, and preliminary analysis [16]. Processing may occur locally on edge devices (e.g., Raspberry Pi, Jetson Nano), remotely via cloud platforms, or both. Edge devices are particularly useful to process high-volume data such as video and audio, reducing bandwidth demands, providing low latency, and ensuring operation under poor connectivity. For instance, edge computing can identify abnormal feeding or feather-pecking behaviors in real time while sending summarized data to the cloud for further analysis. Based on these data, cloud platforms can provide long-term analytics, disease prediction, and visualization dashboards [17]. Advances in AI and affordable HPC in recent years have expanded the capabilities of this layer, enabling intelligent, automated decision-making on farms.

### 2.4. Application Layer

This layer delivers processed information to users, including farm managers, veterinarians, and integrators. It typically takes the form of dashboards, mobile applications, or automated control systems. For example, dashboards can visualize flock health and environmental conditions, while automated controllers can adjust ventilation or lighting based on sensor feedback [18]. The effectiveness of this layer depends on presenting complex data in accessible formats, supporting timely and informed decisions.

Overall, the four-layer IoT architecture provides a flexible framework for poultry systems by integrating sensors, communication networks, and computing platforms into a single unit. Additionally, its success depends on balancing cost, scalability, data reliability, and usability. Continued development of edge computing, energy-efficient sensors, and secure communication protocols will be central to making IoT systems more practical for commercial poultry production.

## 3. IoT Applications in Poultry Production

Modern poultry farms using IoT technology depend significantly on various types of sensors that track environmental conditions and the behavior of birds. These sensors vary in terms of cost, power consumption, precision, and scalability. Their performance is maximized when organized into a closed-loop system. In this setup, sensors in the perception layer send data to edge or cloud platforms for analysis and decision-making, which then control application layer devices such as fans, heaters, feeders, and drinking system. This process can control factors like temperature, harmful gases, lighting, feed, and water flow, ultimately stabilizing environmental conditions, improving animal welfare, reducing labor costs, and enhancing efficiency. Robust closed-loop systems depend on low-latency networking and local computing units such that control actions are timely [15]. Table 1 summarizes representative IoT-enabled poultry studies focused on different tasks, illustrating their evolution from simple environmental monitoring systems to more advanced analytics and decision-support applications. As later sections show, many scenarios such as environmental regulation, automated feeding and watering, and alert systems are closed-loop controls, mostly built on the four-layer architecture discussed in Section 2.

### 3.1. Environmental Sensing and Climate Control

#### 3.1.1. Climate Control and Monitoring

Connected systems are capable of automatically regulating ventilation, heating, and cooling based on sensor data. Sensors in IoT systems can continuously measure temperature, humidity, and gas levels to maintain optimal house conditions [25]. For instance, in an IoT-based poultry farm in Brunei, fans or heaters were automatically activated whenever temperature or air quality went beyond set limits, while alerts were simultaneously sent to managers through SMS or WhatsApp [18]. Such closed-loop control ensures stable conditions (e.g., holding barn temperature at 32–34 °C and 65–75% humidity) while reducing labor and human errors.

#### 3.1.2. Water Quality Monitoring

Both the physicochemical and microbial properties of water are equally important in poultry production. For example, excessive levels of dissolved solids in water negatively impact the feed intake and body weight gain in broilers [26]. Similarly, it is also evident that supplying ionized and cold water can be effective in alleviating heat stress in the birds [27]. IoT-based systems have been developed to monitor and ensure water availability in poultry houses [20,28]. Cloete et al. [19] developed an IoT-capable sensor system for monitoring temperature, flow rate, pH, and oxidation-reduction potential of the water. The system was then able to transmit data wirelessly and notify if the properties reached unsafe levels. Although not originally developed for poultry applications, similar systems can be utilized in poultry houses with minimal modification for continuous water quality monitoring. Several studies have developed IoT-based systems that can detect water flow and leaks in nipple lines [29,30,31]. Although several studies have identified biofilm and bacterial contamination in nipple systems and water lines in poultry production [32,33], direct measurement of these parameters is limited in poultry-specific applications. However, it should be noted that factors affecting biofilms, such as temperature and water flow rate [34], can be readily monitored using existing systems.

### 3.2. Behavior and Welfare Monitoring

#### 3.2.1. Vision-Based Systems

Vision-based systems are among the most widely used technologies in poultry monitoring, largely due to their low cost, non-invasive nature, and the availability of robust deep learning models that allow automation [35,36,37]. Cameras and vision models monitor bird behaviors like activity levels, clustering, and pecking. Advanced image analysis can detect anomalies or distress—for example, a deep learning system identified dead hens on the floor with ~97–100% recall, enabling quick removal to maintain hygiene [38]. Other studies have used cameras capable of multiple imaging modalities: thermal, near-infrared, and depth imaging to detect dead birds based on abnormally low body temperature and lack of movement in caged hens [39,40]. In computer vision-based systems, convolutional neural networks (CNNs) are widely used to detect, track, and classify chicken behaviors with high accuracy, even in complex, crowded environments [41,42,43]. Additional uses include disease detection and prediction [44]. Such systems rely on visual cues and behavioral changes indicative of early disease onset. For example, *E. coli* infection in broiler chickens led to reduced activity levels, as evidenced by visual data analyzed using deep learning [44]. Furthermore, visual data are used to extract posture features that are indicative of diseases such as Newcastle disease and bird flu [45,46]. Although widely used in research, one review of open-access visual datasets found that open-source datasets, especially for commercial settings, remain scarce, thereby slowing the advancement of vision-based systems [47].

#### 3.2.2. Acoustic Systems

Audio sources provide an additional dimension to welfare assessment. Chickens’ vocalizations change in response to stress, hunger, or discomfort, and machine-learning approaches can classify such vocal sounds with an accuracy of approximately 95%, distinguishing normal calls from distress cries or hunger signals [48]. Another study demonstrated that a TinyML model deployed on-farm was able to run continuously on a microcontroller, detecting vocal indicators of stress versus baseline states with ~96% accuracy, thereby providing a low-cost, real-time welfare alert system [49]. Acoustic signals are also indicative of several aspects of productivity, such as weight, feed efficiency, and egg production. Multiple studies have shown that broiler vocalizations are significantly correlated with birds’ age and body weight; as birds age and gain weight, vocalization frequency decreases [50,51,52]. This provides strong evidence that such systems can be used in IoT systems to monitor the welfare status of birds on the farm.

#### 3.2.3. Wearable Systems

Accelerometer-based collars or leg bands offer individual-level behavior tracking. Researchers have used wearable motion sensors to log activities like walking, roosting, and perching frequency for each bird [8]. Such data helps identify outlier birds, for example, an inactive hen in a flock, that may be injured or ill. However, scaling wearables to thousands of birds is challenging. While individual identification systems such as RFID tags have been used in research or small-scale operations, their commercial use remains limited, due to handling and scaling issues [36].

### 3.3. Health Monitoring

#### 3.3.1. Anomaly Detection

IoT and AI enable proactive health management by early detection of unhealthy bird signs. Rather than waiting for clearer signs, forecasting systems can analyze small deviations in normal patterns such as reduced feed intake, reduced movement, or unusual vocalizations and alert farm personnel to check those birds. For instance, Abdoli et al. [53] proposed a predictive platform was proposed that combines wearable sensors and time-series analytics to flag sick birds early. In their approach, accelerometer data from chickens representing tracking behaviors such as dust bathing and preening, were used to classify individuals as healthy or infected. As chickens deliberately infested with parasites showed a distinct reduction in activity, it was classified as an illness indicator using machine-learning models. Such data-driven anomaly detection can prompt isolation or treatment of sick birds before a disease spreads.

#### 3.3.2. Disease Monitoring

Beyond real-time alerts, AI-based analytics help predict disease risks and outcomes. Patterns learned from past data can forecast the likelihood of an outbreak or estimate how an ongoing health issue will affect growth. For example, previous studies have used algorithms like support vector machine (SVM) to identify broilers infected with H5N2 avian influenza with ~99% accuracy. In the present scenario, a dataset developed by [54] stands out as it includes three major infectious diseases—coccidiosis, salmonellosis, and Newcastle disease. Although disease monitoring using a single data source is often accurate, a combination of multiple data sources can provide more insightful information. For example, a sudden rise in ammonia levels with simultaneous coughing sounds and lethargic bird movement is a better indicator of respiratory disease than a single factor. Interconnected systems such as IoT make it feasible to monitor such signals arising from different sources. Other similar systems may provide early warning, alerting farmers to respiratory outbreaks even before visual signs (like nasal discharge or mortality) become evident. Similarly, computer vision can be used to extract features from body posture that indicate illness [46], while thermal cameras might spot fever, indicative of disease onset, by detecting slight temperature elevations in birds [55]. By combining multiple video or image data, acoustic data, and environmental data, AI-driven IoT systems can enhance sensitivity and reduce false alarms in disease detection.

### 3.4. Productivity and Health Monitoring

#### 3.4.1. Automated Weight Tracking

IoT solutions help track bird growth rates and uniformity without manual weighing. Smart scales placed in poultry houses (for example, under feeders or in dedicated weighing stations) log weight data continuously and upload it to the cloud [56]. This provides daily average weight and growth curve information, alerting farmers if birds are not gaining weight as expected. Camera-based weight estimation is another approach: overhead depth cameras or 2D vision combined with AI can estimate body weight by analyzing bird dimensions on screen [57]. Other weight sampling methods also include a physical perch-based weighing rod and vision systems used together [58]. These methods allow frequent, stress-free monitoring of growth progress and enable the detection of stunted individuals that might need attention.

#### 3.4.2. Feed Intake and Conversion

IoT sensors closely monitor feeding behavior to improve feed conversion ratio (FCR). Using load cells on feed bins or vision systems at feeders, the frequency and quantity of the birds’ drinking and feeding behavior can be measured [18,59]. Abrupt drops in intake trigger alerts, indicating possible health issues or equipment faults such as jammed feeders, blocked or dry drinkers. Analyzing intake alongside weight trajectories allows managers to adjust rations or feeder settings to sustain growth. Likewise, persistently high feed intake with limited weight gain may indicate poor FCR in broilers, potentially resulting in economic loss. In addition, AI-based analytics can identify these patterns so nutritionists can intervene, while intelligent systems such as automatic feeders can prioritize underperforming birds, in turn maximizing the welfare and productivity.

#### 3.4.3. Growth Forecasting and Uniformity

With continuous data, producers can forecast when flocks will reach target weight and plan harvest dates more accurately. Machine-learning models have been applied to IoT data to predict growth under different environmental conditions. For example, Kiruthika et al. [60] used a support vector machine (SVM) to predict poultry growth using important IoT-sourced data, including temperature, humidity, light, weight, and water consumption, thereby achieving a 91% accuracy. By accounting for such factors, AI-driven forecasts of market readiness are more precise.

### 3.5. Farm Automation

#### 3.5.1. Smart Environmental Control

IoT-based automation now handles many routine adjustments in poultry houses. Sensors tie into controllers for fans, heaters, misters, and lights to maintain setpoints without human input. In one prototype developed for a broiler house application, temperature, humidity, and air quality were not only monitored but also automatically regulated. When values drifted beyond thresholds, the system activated ventilators or heating lamps to bring conditions back within the target range [18]. This same system also dispatched instant alerts to the farmer’s phone when exceptions occurred, ensuring no critical event goes unnoticed. Automated lighting systems can dim or brighten on schedule to regulate circadian rhythms and growth, and feeding machines can dispense feed at optimal times or quantities. By closing the loop from sensing to automated actions, such IoT setups improve precision and reduce labor, while maintaining environmental parameters within the desired range and controlling fluctuations.

#### 3.5.2. Robotic Applications

IoT integration paves the way for robotics in poultry barns. Autonomous robots can be scheduled or triggered to perform tasks like litter conditioning, cleaning, or health inspections. One innovative system combined IoT sensors with a mobile sanitizing robot to automatically disinfect broiler houses [23]. In this setup, sensors measure litter conditions (e.g., moisture, temperature, pH) and once parameters indicate the need for cleaning, a robot equipped with an ozone sprayer and UV light navigates the barn to neutralize pathogens and pests. This reduces human exposure to chemicals and ensures consistent biosecurity. Similarly, prototype “inspection robots” like the RoboChick developed in the UK by Demmers et al. [61] roam through flocks with cameras and environmental sensors, checking for issues such as malfunctions or sick birds and reporting data back via the IoT network. While still emerging, these robotic integrations are useful in handling repetitive tasks and improving overall farm efficiency. Although studies on robotic applications in poultry houses have shown avoidance responses in birds, such behavior declines over time due to habituation [62,63]. Additionally, research has shown that birds exhibit lower or similar behavioral responses, such as avoidance distance, in the presence of robots than human handlers do [64]. Another study found that robots can promote movement and exploratory behavior in broilers, potentially improving their welfare [65]. However, there is a need to assess whether such applications affect the birds’ welfare in the long term.

## 4. Data Processing and Analytics

In poultry IoT systems, massive data streams flow from on-farm devices like cameras, microphones, environmental sensors, and load cells. The core challenge lies in processing this volume of information quickly and accurately so that useful insights are delivered in a timely and efficient manner. One effective strategy to manage the load is deploying AI models on edge devices (such as smart cameras or sensor hubs), enabling local inference on the device itself and sending only distilled insights to cloud platforms for further analysis.

Recent poultry-domain studies demonstrate the value of these edge and hybrid architectures. For instance, Cakic et al. [22] ported a Faster R-CNN model to an edge computing platform and successfully detected chickens with high precision under real-time constraints. Similarly, Tong et al. [24] implemented a compact deep learning model called FCOS-Lite on an intelligent camera sensor, achieving a mean average precision (mAP) of 95.1% with real-time inference exceeding 20 frames per second. To further reduce the computational burden, some systems adopt zone-based tracking, restricting behavior detection to defined regions (e.g., around feeders or drinkers) and focusing on group-level patterns to simplify analysis [66].

Beyond computer vision, acoustic sensing is also being leveraged at the edge. A recent TinyML-based system, for example, classified chicken vocalizations into stress, hunger, or baseline states with roughly 96% accuracy on microcontroller hardware, providing a low-power means for continuous welfare monitoring [49]. In such designs, the edge device filters, aggregates, interprets, and stores sensor data locally [49]. Similar acoustic data can be uploaded to the cloud later, enabling growers to make data-driven decisions [67].

To build an IoT-enabled poultry house that generates a large volume of data [68], it is necessary to aggressively optimize models and compress data to enable on-device inference [69]. Techniques like model pruning, quantization, and neural architecture search are commonly applied to shrink model sizes and reduce complexity, ensuring that real-time analysis can run smoothly on resource-constrained hardware [70,71]. Deployed models must also generalize across farm conditions with varying lighting, camera angles, and flock densities. Latency is another important factor: many poultry IoT systems require that inferences be completed within tens to a few hundred milliseconds to effectively close control loops, for example, adjusting ventilation or triggering alarms based on sensor readings [72]. Furthermore, low-cost sensors commonly used in poultry IoT systems suffer from errors due to sensor drift, adverse farm conditions, and environmental factors, necessitating proper calibration to maintain accuracy and reliability over time [73].

## 5. Challenges

### 5.1. Scalability

Poultry operations range from highly integrated commercial complexes to small and medium farms, even backyard poultry coops, and this diversity complicates uniform deployment. Large houses can justify dense sensor grids, dedicated networking, and on-site computing, whereas smaller farms often face budget limits and intermittent connectivity, leading to sparse instrumentation and greater reliance on cloud-only workflows. Data volume scales rapidly with cameras and microphones, pressuring backhaul and storage; wiring is reliable but costly at barn scale, while wireless reduces installation time but can suffer interference due to metallic structures, attenuation from dust and moisture, and coverage dead zones [11,15,74]. Ongoing maintenance adds another layer: gas and particulate sensors drift and foul in barn air, requiring calibration and protective housing; cameras require lens cleaning and lighting management; and firmware must be kept current to avoid downtime [14,21]. Fault tolerance is also a design priority as redundant nodes, watchdogs, and local buffering limit data loss during outages, and fail-safe modes preserve ventilation or alarms if higher layers fail [17]. Finally, power management and hardware ruggedness matter as the edge devices must operate within tight energy budgets and withstand heat, humidity, dust, and corrosive gases without frequent replacement [15,25].

### 5.2. Data Privacy and Security

Connected barns expand the attack surface. Threats include spoofed sensor data that could mislead automated control, unauthorized access to cameras or historical records, and denial-of-service events that disrupt alerts or actuation [13,14]. Mitigations span multiple layers: resilient access control and network segmentation; authenticated time-stamped telemetry; and encryption in transit and at rest, including on removable media used for edge buffering [14]. Lightweight, on-device anomaly detection can flag improbable traffic patterns or sensor states, isolating compromised nodes at the edge before data propagate [13]. Governance choices also play a vital role. Blockchain-backed logs can provide tamper-evident audit trails for biosecurity and welfare compliance, while federated learning allows model training across farms without centralizing raw data, reducing privacy exposure [12]. Clear policies on data ownership, retention, and sharing, especially for video/audio are necessary when multiple parties (growers, integrators, vendors) interact and should be documented alongside incident response procedures [12,14].

### 5.3. Interoperability and Standardization

Heterogeneous devices, protocols, and vendor ecosystems limit plug-and-play integration at farm scale. Differences in messaging stacks (e.g., MQTT variations), data schemas, and time-sync practices can fragment deployments, complicate cross-house analytics, and raise lifecycle costs when components are upgraded [11,15]. Sparse or proprietary application programming interfaces (APIs)and nonstandard metadata impede model portability and benchmarking across sites. To reduce vendor lock-in, projects benefit from adopting open interfaces and shared ontologies for poultry events (feeding, perching, mortality), sensor health, and actuation commands. Time synchronization and consistency, unit-aware metadata improve data fusion across modalities (video, audio, environmental). Edge-to-cloud contracts that specify message formats, quality-of-service, and back-pressure behavior help prevent silent data loss under congestion [16,17].

### 5.4. Cost and Adoption Barriers

Upfront investment in sensors, networking, edge compute, and integration is a hurdle especially for smaller farms while perceived risks (technology obsolescence, maintenance burden) slow adoption. Demonstrating return on investment requires credible links from measurements to outcomes fewer losses from heat stress, improved uniformity, better feed conversion, or reduced labor. Low-cost nodes show promise, e.g., Pereira et al. [21] reported >0.90 correlation between inexpensive and reference environmental sensors at a fraction of the cost, but long-term durability and calibration demands must be budgeted. However, harsh conditions in poultry houses lead to data loss, systematic errors, and long-term hardware degradation [75], which may slow adoption. Furthermore, most developed systems are prototyped and used in research facilities, with only a few available as commercial tools [35]. In addition to the cost and availability, usability is also important to consider. Many components used in IoT systems have high maintenance and calibration requirements [75,76] and may be considered obstacles to adoption. Finally, there is an evidence gap as real-world case studies are needed to quantify welfare and productivity gains under real-world commercial conditions and to clarify which bundles of sensing, analytics, and automation deliver the best value [11,17].

## 6. Impacts and Outcomes

IoT-backed systems in poultry farming have demonstrated notable gains in efficiency. For example, smart environmental control can optimize conditions like temperature and ventilation, improving feed conversion and reducing wasted resources [18]. A robotic litter sanitation system showed how automation can cut energy use by optimizing movement paths—a back-and-forth navigation pattern minimized turning, thereby lowering power consumption during litter decontamination [23]. Similarly, integrating AI with IoT (e.g., computer vision at the edge) can increase the FCR and reduce mortality, leading to more efficient production overall [22]. Advanced predictive models using farm sensor data (temperature, humidity, etc.) have been used to avoid resource waste while boosting growth—in one case, machine learning-based modeling improved poultry growth and feed usage efficiency, promoting more sustainable farming practices [60]. In short, IoT implementations help farmers do more with less by tightening control over inputs and environmental parameters.

### 6.1. Labor and Cost Savings

Automation through IoT yields substantial labor and cost benefits in poultry operations. An IoT-based smart farm prototype in Brunei, for instance, reduced the need for manual labor by automating routine tasks like climate control and feeding, which not only cut manpower requirements but also improved overall farm efficiency [18]. Another clear example is automatic mortality detection: using cameras and AI to detect dead birds can save time and labor compared to farm workers manually inspecting large flocks [38]. This type of automated monitoring enables the timely removal of dead chickens without constant human oversight, streamlining operations. These technologies not only reduce labor costs but also decrease human error and oversight costs. Additionally, examples like low-cost IoT/robotics approach for litter sanitation by Cechinel et al. [23] and the low-cost IoT-based environmental monitoring system developed by Pereira et al. [21] have the potential to make the technology accessible to poultry producers without prohibitive expense. Overall, automating monitoring and control tasks translates to fewer hours on manual checks and interventions, bringing down labor costs and improving the cost-effectiveness of poultry management.

### 6.2. Welfare Improvements

A major driver for IoT in poultry is improving animal welfare through constant, gentle monitoring and early issue detection. Continuous monitoring of chickens’ behavior and health using sensors allows farmers to detect problems at an early stage. This leads to faster interventions that reduce stress and disease in the flock. For example, systems that analyze chicken vocalizations or activity can identify signs of distress or illness sooner, enabling treatment or environmental adjustments before issues escalate [77]. Early detection of sick birds is important as it prevents the spread of disease and large-scale losses, ultimately safeguarding flock welfare and farm productivity [8]. Overall, IoT deployments help maintain optimum conditions (temperature, air quality, etc.), leading to less stress (avoiding heat stress, poor air quality or frequent handling) and prompt care (alerting farmers to issues like disease outbreaks or water/feed shortages). In summary, these technologies improve welfare by ensuring chickens are kept in comfortable conditions and by enabling early, targeted interventions when problems arise.

### 6.3. Potential Risks and Consequences

Despite the benefits, IoT adoption in poultry comes with challenges and potential unintended effects. Technical feasibility and cost can be a limiting factor. For example, while wearable sensors can monitor individual animals, equipping every bird with a device is often not practical or cost-effective in large flocks as the initial investment cost and maintenance costs can quickly multiply with increase in number of birds. There is also the risk of over-reliance on technology. For example, if an automated system fails or misidentifies conditions, issues could go unnoticed. Currently, vision systems are not yet 100% reliable in commercial settings due to the sheer number of birds and the presence of equipment in large scale poultry houses, resulting in occlusion and highly variable lighting which might lead to inaccurate data. Another concern is data security and system resilience. IoT devices introduce cyber-security vulnerabilities and data privacy concerns and researchers have noted the need for robust IoT architectures to prevent hacking or data breaches in connected farms [11]. Similarly, network or power failures could disrupt automated systems, potentially impacting animal care if there are no manual fail-safes. There are also human safety considerations: traditional practices like manual pesticide spraying in barns expose workers to health risks, and while robotic IoT solutions can remove humans from harm’s way [23], they must be implemented carefully to ensure they operate safely around both animals and workers. Unintended consequences such as technology-induced stress, for example, if sensors or robots are intrusive to the birds, should be monitored. However, technologies like wearable sensors can be used to learn behavioral patterns of the birds for a short duration of time [53]. In summary, to fully utilize IoT to the collective advantage, technical, financial, and ethical risks need to be managed through thoughtful design, such as secure networks, backup systems, and animal-centric engineering.

## 7. Research Gaps and Future Directions

Most IoT deployments in poultry are validated in small pilots and either rely on a single sensing modality or treat multiple sensors as separate, non-integrated channels that drive different actuators. This architecture is fragile under real barn conditions, where lighting, dust, occlusion, and noise vary throughout the day, and it reduces confidence in closed-loop control. Future work should prioritize multimodal sensor fusion, so signals corroborate and complement one another. Time-synchronized video, audio, environmental readings, load-cell events, and, where feasible, lightweight wearables should be fused at the data, feature, or decision level with shared timestamps and calibration. For example, vision-only behavior detection degrades when birds or feeders occlude the camera view. Adding a second camera angle and auxiliary cues, such as wearables or acoustic indicators, can recover missed events and suppress false alarms. Fused pipelines provide cross-validation and maintain accuracy when any single channel underperforms.

Effective implementation also requires resource-efficient edge devices that generate useful insights and support real-time decisions without sending large volumes of raw data to the cloud. This reduces network load and improves data security. Recent advances in micro-electronics and low-power computing have made compact edge deployments practical, and several studies have already demonstrated edge inference in poultry settings [17,22]. Complementary progress in lightweight models further strengthens feasibility, for example, TinyML classifiers for vocalizations and compact detectors for health or behavior, both running at real-time speeds on constrained hardware [24,49]. Power remains a bottleneck for wearables and distributed nodes. Therefore, pairing low-power devices with adaptive functionality, including sleep modes during rest periods and event-driven wakeups, is recommended [49].

Although technical performance is important, it is equally important to validate whether data from these systems accurately reflect health, welfare, and productivity outcomes. For example, although several studies have shown correlations, most systems are validated under controlled conditions, with limited multi-farm or long-term evaluations [37,38,39]. Therefore, interpreting data collected by IoT systems in commercial or real-world settings remains a key gap. Furthermore, barns are dusty, humid, and often high in ammonia, which is harsh on electronics. Long-term feasibility of general-purpose components should also be evaluated under poultry-house conditions, and purpose-built, low-cost hardware should be explored for sustained use. A modular pipeline that supports over-the-air updates and self-calibration is essential to enable systems to evolve without full hardware replacement. This reduces maintenance burden and avoids costly downtime in commercial operations.

Finally, adoption depends primarily on economics, regardless of how well the technology performs in research conditions. Farm-scale studies should therefore quantify return on investment, including effects on feed conversion, labor, and losses avoided, and should explore technologies from other domains. Animal welfare and data privacy must be integral to design. As technology density increases, so do failure modes and exposure, which makes secure data handling, clear ownership and retention policies, and well-tested fail-safe behaviors necessary for responsible deployment.

## 8. Conclusions

Interconnected systems like IoT and AI together can reshape poultry production by turning continuous sensing into timely, actionable decisions that improve efficiency, reduce labor, and support bird welfare. A layered architecture with edge intelligence enables real-time monitoring and closed-loop control, while emerging technologies such as edge computing, multimodal fusion and lightweight models promise broader impact across diverse farm conditions. Remaining barriers include farm-scale validation, durable and self-calibrating hardware, shared standards for interoperability, clear return of investment, and strong practices for data security and welfare. Progress will depend on coordinated trials with growers and integrators, modular systems that can evolve without full replacement, and tight coupling of sensing with actuation and robotics. With these elements in place, IoT-enabled, AI-driven systems can become dependable, affordable, and welfare-oriented tools for commercial poultry production.

## Figures and Tables

**Table 1:** Summary of representative IoT-based systems applied in poultry production.

Task	Perception Layer	Network Layer/Communication Protocols	Processing Layer	Application Layer	References
Water quality monitoring	Flow sensor, temperature sensor, conductivity sensor, pH sensor, electrodes	ZigBee	Microcontroller	Buzzer, LCD display	Cloete et al. (2016) [19]
Environmental monitoring	Light, temperature and humidity sensor	Wi-Fi communication, Cloud synchronization via Firebase	Single board computer (SBC)	Web-based dashboard	Manshor et al. (2019) [20]
Environmental and feed level monitoring	Temperature and humidity sensor, air quality sensor, photoresistor	Wi-Fi communication via ESP8266, RESTful web services	SBC	Web-based dashboard, fan exhaust system, LCD display, SMS and WhatsApp notification	Hambali et al. (2020) [18]
Environmental monitoring	Temperature and humidity sensor, Gas sensor, Photoresistor	Wi-Fi communication,	Microcontroller Unit	Android mobile application	Pereira et al. (2020) [21]
Disease monitoring	Accelerometers	Wireless data transmission, Cloud based data transfer	Cloud based data processing using deep learning	Real-time health classification dashboard	Ahmed et al. (2021) [8]
Edge AI-based poultry monitoring	Cameras, environmental sensors	Ethernet, Long Range (LoRa), Bluetooth Low Energy (BLE), and 4G cellular	Edge AI devices, High performance computing (HPC) cluster, Cloud based IoT platform	Web-based dashboard, mobile application	Cakic et al. (2023) [22]
Autonomous litter sanitation	Poultry litter sensors, Cameras, Ozone sensors	Wi-Fi, Robot middleware communication	SBC, PC	Dashboard with decision support	Cechinel et al. (2024) [23]
Health monitoring	AI-enabled camera, CMOS sensor	USB connection	AI-enabled camera, Deep learning architecture, graphic processing unit (GPU)	Visualization of chicken health results	Tong et al. (2024) [24]

## Data Availability

No new data were created or analyzed in this study. Data sharing is not applicable to this review article.
